# A Predictive Model for Thiamine Responsive Disorders Among Infants and Young Children: Results from a Prospective Cohort Study in Lao People's Democratic Republic

**DOI:** 10.1016/j.jpeds.2024.113961

**Published:** 2024-05

**Authors:** Taryn J. Smith, Charles D. Arnold, Philip R. Fischer, Indi Trehan, Laurent Hiffler, Dalaphone Sitthideth, Rebecca Stein-Wexler, Jay Yeh, Kerry S. Jones, Daniela Hampel, Daniel J. Tancredi, Michael A. Schick, Christine N. McBeth, Xiuping Tan, Lindsay H. Allen, Somphou Sayasone, Sengchanh Kounnavong, Sonja Y. Hess

**Affiliations:** 1 Institute for Global Nutrition and Department of Nutrition, University of California Davis, Davis, CA; 2 Department of Pediatric and Adolescent Medicine, Mayo Clinic, Rochester, MN; 3 Pediatrics Department, Sheikh Shakhbout Medical City, Abu Dhabi, United Arab Emirates; 4 Khalifa University, Abu Dhabi, United Arab Emirates; 5 Departments of Pediatrics, Global Health, and Epidemiology, University of Washington, Seattle, WA; 6 Lao Friends Hospital for Children, Luang Prabang, Lao People's Democratic Republic; 7 Cellular Nutrition Research Group, Lagny sur Marne, France; 8 Lao Tropical and Public Health Institute, Vientiane Capital, Lao People's Democratic Republic; 9 Department of Radiology, University of California Davis Health System, Sacramento, CA; 10 Department of Pediatrics, Division of Cardiology, University of California Davis Health System, Sacramento, CA; 11 Nutritional Biomarker Laboratory, MRC Epidemiology Unit, University of Cambridge, Cambridge, UK; 12 USDA-Agricultural Research Service Western Human Nutrition Research Center, Davis, CA; 13 Department of Pediatrics, University of California Davis Health System, Sacramento, CA; 14 Emergency Medicine, University of California Davis Health System, Sacramento, CA

**Keywords:** beriberi, thiamine deficiency, thiamine deficiency disorders, critical illness, cardiac distress, respiratory distress, encephalopathy, Southeast Asia

## Abstract

**Objective:**

To develop a predictive model for thiamine responsive disorders (TRDs) among infants and young children hospitalized with signs or symptoms suggestive of thiamine deficiency disorders (TDDs) based on response to therapeutic thiamine in a high-risk setting.

**Study design:**

Children aged 21 days to <18 months hospitalized with signs or symptoms suggestive of TDD in northern Lao People’s Democratic Republic were treated with parenteral thiamine (100 mg daily) for ≥3 days in addition to routine care. Physical examinations and recovery assessments were conducted frequently for 72 hours after thiamine was initiated. Individual case reports were independently reviewed by three pediatricians who assigned a TRD status (TRD or non-TRD), which served as the dependent variable in logistic regression models to identify predictors of TRD. Model performance was quantified by empirical area under the receiver operating characteristic curve.

**Results:**

A total of 449 children (median [Q1, Q3] 2.9 [1.7, 5.7] months old; 70.3% exclusively/predominantly breastfed) were enrolled; 60.8% had a TRD. Among 52 candidate variables, those most predictive of TRD were exclusive/predominant breastfeeding, hoarse voice/loss of voice, cyanosis, no eye contact, and no diarrhea in the previous 2 weeks. The area under the receiver operating characteristic curve (95% CI) was 0.82 (0.78, 0.86).

**Conclusions:**

In this study, the majority of children with signs or symptoms of TDD responded favorably to thiamine. While five specific features were predictive of TRD, the high prevalence of TRD suggests that thiamine should be administered to all infants and children presenting with any signs or symptoms consistent with TDD in similar high-risk settings. The usefulness of the predictive model in other contexts warrants further exploration and refinement.

**Trial registration:**

Clinicaltrials.gov NCT03626337.

Thiamine deficiency disorders (TDDs) include the broad spectrum of overlapping clinical presentations associated with thiamine (vitamin B1) deficiency across the life course, including the classic forms of beriberi among infants and young children.^1^^,^^2^ TDD and beriberi continue to be important causes of morbidity and mortality among breastfed infants in South^3^, ^4^, ^5^, ^6^ and Southeast Asia,^7^, ^8^, ^9^ including Lao People's Democratic Republic,^10^^,^^11^ and are largely attributable to maternal thiamine deficiency and resulting insufficient human milk thiamine due to diets based on thiamine-poor polished white rice.^1^^,^^12^

Due to the broad and nonspecific clinical presentations of thiamine deficiency, ranging from cardiogenic shock to neurological manifestations, and the observation that infantile TDD often presents in the context of acute infections or other conditions with overlapping clinical signs and symptoms,^13^ TDD may go unrecognized by health care providers, particularly in the absence of specific diagnostic tests. Due to these uncertainties, there is likely a high rate of misdiagnosis among at-risk populations leading to missed or delayed treatment, with fatal consequences or permanent neurologic sequelae.^7^^,^^14^^,^^15^ However, based on observations that clinical signs resulting from thiamine deficiency respond rapidly to thiamine administration,^2^ it is possible to identify thiamine responsive children among those with possible TDD (based on their presenting signs and symptoms) and explore which clinical signs and risk factors best predict thiamine responsiveness, to help determine those who will benefit from thiamine treatment.

Therefore, the aim of this study was to develop a predictive model for thiamine responsive disorders (TRD) among infants and young children hospitalized with possible TDD in a high-risk setting, based on the clinical signs and other predictors that distinguished those who responded positively to thiamine administration from those who did not respond. Specifically, the objective was to develop two versions of the predictive model, 1 to include only information available in low resource settings, and 1 for high resource settings.

## Methods

### Study Design, Setting, and Participants

Infants and young children 21 days-<18 months of age who were hospitalized at the Lao Friends Hospital for Children in Luang Prabang, northern Lao People’s Democratic Republic with signs and symptoms consistent with TDD were enrolled into a prospective cohort study between June 2019 and December 2020 (registered at clinicaltrials.gov as NCT03626337). The study protocol has previously been described in detail.^16^ Hospitalized children were eligible if they presented with at least 1 of the following signs and symptoms consistent with TDD: hepatomegaly (liver edge >2 cm below the right costal margin), edema, tachypnea (respiratory rate >60/min for infants 3-8 weeks old; >50/min for infants 2-11 months old; >40/min for children 12-18 months old), tachycardia (heart rate >160/min for under 12 month olds; >120/min for children 12-18 months old), oxygen saturation <92%, respiratory distress (chest indrawing or nasal flaring), refusal to breastfeed or consume infant formula or food for >24 hours, repetitive or recurrent vomiting with no other apparent cause (>3 times in past 24 hours), persistent crying not relieved by soothing or feeding with no obvious other cause, hoarse voice/cry or loss of voice, nystagmus or other unusual eye movements, muscle twitching, loss of consciousness, convulsion, opisthotonos/abnormal posturing, and acute/flaccid paralysis.

### Data Collection Procedures

Children enrolled into the study were treated by hospital physicians following usual hospital practices. Once children were screened and identified as eligible and informed consent obtained from at least 1 primary caregiver, dedicated study physicians and nurses performed a detailed baseline physical exam and echocardiogram and obtained a venous blood sample. All enrolled children were treated with 100 mg parenteral thiamine (intravenous when possible) daily for a minimum of 3 days, alongside any other treatments deemed necessary by the treating physicians. Once the initial thiamine dose was administered, physical examinations were repeated after 4, 8, 12, 24, 36, 48, and 72 hours. One cranial ultrasound was performed, typically within the first 12 hours, and echocardiograms were repeated at 24 and 48 hours. For the echocardiogram, 2 second video clips of the heart in apical four chamber, parasternal long-axis and parasternal short-axis views were captured, along with still images for the measurement of left ventricular dimensions in diastole and systole M-mode, E-point septal separation in the parasternal long-axis, fractional shortening and tricuspid annular plane systolic excursion. For the cranial ultrasound, six standard coronal and five standard sagittal images were captured, with additional images of the putamina, caudate nuclei, and thalamus. All echocardiograms and cranial ultrasounds were reviewed by emergency physician sonographers at the University of California Davis, and, if any abnormalities were identified, scans were reviewed by a pediatric cardiologist and pediatric radiologist, respectively. For quality assurance, 10% of randomly selected echocardiograms and cranial ultrasounds were reread by the pediatric cardiologist and radiologist, respectively.^16^ When ultrasounds suggested abnormalities due to potential thiamine deficiency, children were invited for follow-up examinations after 2-4 weeks for abnormal echocardiograms and 6 weeks for abnormal cranial ultrasounds, although these follow-up findings were not incorporated into the TRD predictive model or presented here.

At the 4, 8, 12, 24, 36, 48, and 72 hour assessments, the child's clinical status was assessed using a five-point scale (seems worse, no change, seems a little bit better, seems a lot better, seems recovered), and whether the child was well enough to be discharged from hospital or needed to remain for ongoing treatment. If a child's health stabilized before 48 hours, caregivers were asked if they would be willing to remain at the hospital until 48 hours. If a child remained in hospital for >72 hours, no further data were collected until a final physical examination at discharge. At discharge, data were collected on the final primary diagnosis and any secondary diagnoses by the treating hospital physicians, any treatments and medications administered during the hospital stay, and if the child and/or their mother were provided with oral thiamine supplements to continue after discharge.

Prior to thiamine administration, a 5 mL venous blood sample was collected using 6 mL evacuated EDTA-coated tubes (BD vacutainer, Becton, Dickinson & Co., NJ, USA). Immediately after collection, lactate concentration (StatStrip Lactate, Nova Biomedical, Waltham, MA, USA) and complete blood count (BC-3000 Plus Auto Hematology Analyzer, Mindray Biomedical Electronics, Shenzhen, China) were determined. Whole blood was aliquoted into amber microcentrifuge tubes and stored at −80°C. Blood samples were then centrifuged at 3200 rpm (approximately 1202 x g) for 10 minutes, and the plasma and buffy coat aliquoted separately and stored at −80°C. Erythrocytes were washed 3 times in physiological saline (0.9% NaCl), centrifuged at 4000 rpm (approximately 1878 x g) for 10 minutes each time, and stored in amber microcentrifuge tubes at −80°C.

Whole blood thiamine diphosphate (ThDP) and erythrocyte transketolase activity coefficient (ETKac) were analyzed at the Nutritional Biomarker Laboratory, University of Cambridge, UK. Whole blood ThDP was measured using the thiochrome reaction coupled with high-performance liquid chromatography fluorescence detection.^17^ The ETK activity assay was performed by UV spectrophotometry.^18^ Although there is currently no consensus on the cut-off for thiamine deficiency as measured by ThDP, for the present analysis ThDP <95 nmol/L was used to categorize children with low thiamine status.^19^ An ETKac >1.25 was used to indicate a high risk of thiamine deficiency.^19^^,^^20^ To assess baseline thiamine biomarker status, children who were known to have received thiamine prior to the blood draw at either an external health center or at the hospital, and those who had free thiamine concentration greater than the 90th percentile of the study sample, had their biomarker data excluded from the biomarker analysis. C-reactive protein and alpha-1-acid-glycoprotein were analyzed by enzyme-linked immunosorbent assay (VitMin Lab, Willstaett, Germany).^21^

From breastfeeding mothers, 1 single, full breast expression was collected from the fuller breast using an electronic hospital-grade breast pump (Symphony Pump, Medela AG, Baar, Switzerland). Samples were mixed well before aliquoting and storing at −80°C. Total thiamine concentration was determined by ultraperformance liquid chromatography tandem mass spectrometry (USDA Western Human Nutrition Research Center, Davis, CA, USA).^22^

During the hospital stay, a broad range of sociodemographic, dietary and clinical information was collected with the use of structured questionnaires on potential risk factors for thiamine deficiency.^16^ Each child's recumbent length and weight were assessed following standard protocols.^23^ All data were entered electronically into Samsung tablets (Samsung Galaxy Tab. 3V, Seoul, South Korea) with the use of SurveyCTO (Dobility, Cambridge, Massachusetts, USA).

### Determination of TRD Status

Information collected from enrolled children over the duration of the hospital stay was collated into a case report^24^ that was generated for each child with ≥12 hours of observation and reviewed by three pediatricians with expertise in tropical medicine, including beriberi. The pediatricians independently reviewed each case report, searching for evidence of a rapid response that could be attributable to thiamine administration, while also considering the likelihood of thiamine deficiency as a predisposing factor. This entailed careful consideration of the child's age, breastfeeding status, and maternal signs that may be suggestive of thiamine deficiency and presenting symptoms, changes in symptoms and vital signs over 12-72 hours, and specifically rapid changes within 4-12 hours of thiamine administration, other comorbidities that may have explained the presenting symptoms and clinical course, and other important factors such as ultrasound findings. The pediatricians independently assigned each child to 1 of 4 categories (classic beriberi, probable TRD, possible TRD, not likely TRD) based on all the information compiled in the case reports. Where there was disagreement (when separate reviewers classified a case as probable TRD and not likely TRD) or no agreement (each reviewer assigned a case a different TRD score), case reports were discussed as a group to seek consensus. For use in the clinical prediction model, the four-point TRD scores were collapsed to TRD (classic beriberi and probable TRD) and non-TRD (possible TRD and not likely TRD).

### Statistical Analysis

Original sample size calculations assumed a TRD prevalence of approximately 10% to 20%, two-sided testing with alpha = 0.05, and variance inflation factors of 1.88, 1.25, and 1.25 to account for model selection, loss due to blood collection failures, and loss to follow-up, respectively. Therefore, a target enrollment of 662 was calculated to provide at least 90% power to develop a model with a discriminative capacity corresponding to an area under the receiver operating characteristic curve (AUROC) of 0.70. Due to lower than anticipated enrollment rates but higher TRD prevalence than used in the sample size calculation, the achieved sample size of 449 provided >99% power to detect an AUROC of 0.70. All children with ≥12 hours of follow-up observation data were included in the analyses.

Prior to analysis, a statistical analysis plan was developed and published.^24^ Overall TRD status agreement across all three pediatricians was assessed by absolute agreement rate and Spearman-Brown adjusted reliability statistics. Model development proceeded systematically through Steyerberg's checklist of steps for developing and validating clinical prediction rules.^25^ Analysis followed a three-staged approach to identifying candidate risk factors^24^ and selecting from among them. In the first stage, we identified risk factors that could be reliably and validly measured early in hospitalization in low resource settings and that are plausibly associated with TRD status. In the second stage, logistic regression models were developed using TRD score as the dependent variable with elastic net regression used to select independent variables. After the clinical prediction model was developed, 10-fold cross-validation was used to estimate model performance as measured by empirical AUROC. A second version of the predictive model was developed by incorporating more sophisticated clinical diagnostic assessments, such as biomarkers and ultrasounds, which are available in high resource settings. In the last stage, analysis was repeated according to specific sensitivity analysis scenarios to assess the robustness of the initial model. A prespecified sensitivity analysis was performed including participants who died within 72 hours of enrollment as non-TRD, as it could not be determined if thiamine deficiency was a contributing factor to the death. In addition, several post-hoc sensitivity analyses were performed, including: (1) excluding participants who received thiamine treatment prior to arrival at the hospital; and (2) excluding participants who were not assigned a consensus TRD classification during initial pediatrician review.

## Results

**Figure 1:**
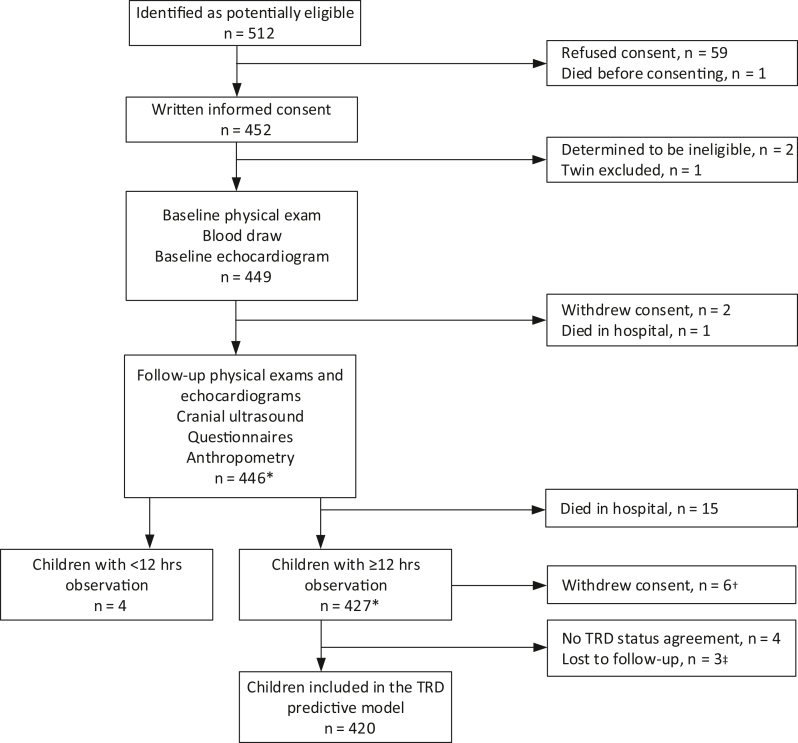
Flowchart of hospitalized children's eligibility, enrollment, and data collection. ∗ Sample size for different assessments may vary. ^†^ Withdrew consent for further data collection during hospitalization but with ≥12 hours of observation so included in analyses. ^‡^ Children discharged from hospital expected to die; not included in TRD model analysis.

A total of 512 children were identified as potentially eligible for participation in the study, of whom 449 completed the baseline assessments (Figure 1). Four children had <12 hours of follow-up observation data, 2 withdrew consent prior to any follow-up assessments, and 16 children died in hospital. Therefore, the pediatricians reviewed case reports for 427 children, and a consensus TRD status was reached for 423 children, with a high level of reviewer agreement (99%; Table I, online; available at www.jpeds.com). Ninety-two children (21.8%) were classified as classic beriberi, 165 (39.0%) as probable TRD, 143 (33.8%) as possible TRD, and 23 (5.4%) as not likely TRD. Thus, 60.8% were classified as TRD and 39.2% as non-TRD. Three children were discharged from hospital expected to die and not included in the predictive model, leaving 420 children included in the model.

The baseline characteristics and clinical profiles of all children are shown in Table II and by TRD status in Table III. Median (Q1, Q3) age of the children was 2.9 (1.7, 5.7) months, 60.8% were male and 70.3% were exclusively or predominantly breastfed. Mean ± SD maternal age was 24.8 ± 6.5 years, 92% of women reported following traditional postpartum food restrictions, whereby many foods except white rice were excluded from the diet for 1-2 months postpartum (previously described in detail^26^), and 34% reported tingling in the fingers or feet in the previous 2 weeks. Parents reported children were ill for a mean of 4.8 ± 8.5 days prior to hospitalization and difficulty breathing was the most common reason for taking the child to the hospital. The majority of children presented with signs and symptoms consistent with cardiac beriberi, with respiratory distress (70.8%), tachycardia (61.7%), and tachypnea (45.2%) being the most common. Few children presented with signs and symptoms consistent with neurological beriberi. Tachypnea, tachycardia, respiratory distress, hoarse voice/loss of voice, persistent crying with no obvious cause, and hepatomegaly were significantly more common among TRD children compared with non-TRD children (Table III).

**Table II:** Baseline characteristics and clinical profile of infants and young children presenting to hospital with thiamine deficiency disorders (n = 449)

Child characteristics	n (%), median (Q1, Q3), or mean ± SD
Male	273 (60.8)
Age (months)	2.9 (1.7, 5.7)
Exclusively or predominantly breastfed∗	315 (70.3)
Stunted†	136 (32.7)
Wasted†	47 (11.4)
Underweight†	119 (28.6)
**Clinical profile of children**	
Duration of illness prior to hospitalization (days)	4.8 ± 8.5
Reasons for caregivers bringing child to hospital‡	
Difficulty breathing	297 (66.3)
Fever	243 (54.2)
Coughing	235 (52.5)
Signs and symptoms at hospital admission§	
Hepatomegaly (palpable >2 cm below right costal margin)¶	139 (31.0)
Edema	9 (2.0)
Tachypnea∗∗	203 (45.2)
Tachycardia∗∗	277 (61.7)
Oxygen saturation <92%	112 (24.9)
Respiratory distress	318 (70.8)
Refusal to feed for >24 hrs	113 (25.2)
Repetitive/recurrent vomiting (≥3 times in 24 hrs)	66 (14.7)
Persistent crying with no obvious cause	177 (39.4)
Hoarse voice/loss of voice	179 (39.9)
Nystagmus/abnormal eye movements	17 (3.8)
Muscle twitching	13 (2.9)
Loss of consciousness	30 (6.7)
Convulsion	40 (8.9)
Opisthotonos/abnormal posturing††	11 (2.4)
Acute/flaccid paralysis††	2 (0.4)

∗ Predominant breastfeeding defined as breastfeeding with the addition of certain liquids (water, water-based drinks, fruit juice).

† Stunting, wasting and underweight defined as length-for-age, weight-for-length, and weight-for-age z scores < −2 SD, respectively.

‡ Multiple responses could be given.

§ Eligibility criteria for enrollment into study.

¶ n = 121 (26.9%) not recorded.

∗∗ Age specific cut-offs: respiratory rate >60/min for 3-8 weeks, >50/min for 2-11 months, >40/min for 12-18 months; heart rate >160/min for <12 months, >120/min for 12-18 months.

†† n = 121 (26.9%) not recorded.

**Table III:** Characteristics of infants and young children and clinical, ultrasonography, and laboratory profiles by thiamine responsive disorder status determined by pediatrician consensus (n = 420)∗

	TRD (n = 256)	Non-TRD (n = 164)	*P* Value
Male	153 (59.8)	104 (63.4)	.454
Age			
<3 months	145 (56.6)	69 (42.1)	.003
3-6 months	78 (30.5)	29 (17.7)	.003
>6 months	33 (12.9)	66 (40.2)	.003
**Signs and symptoms at hospital admission**†			
Hepatomegaly (palpable >2 cm below right costal margin)‡	87 (46.8)	38 (32.2)	.012
Edema	7 (2.7)	2 (1.2)	.436
Tachypnea§	131 (51.2)	53 (32.3)	<.001
Tachycardia¶	181 (70.7)	78 (47.6)	<.001
Oxygen saturation <92%	69 (27.0)	34 (20.9)	.158
Respiratory distress	198 (77.3)	99 (60.4)	.001
Refusal of feed for >24 hrs	66 (25.8)	40 (24.4)	.749
Repetitive/recurrent vomiting (≥3 times in 24 hrs)	88 (34.4)	62 (37.8)	.621
Persistent crying with no obvious cause	119 (46.5)	45 (27.4)	<.001
Hoarse voice/loss of voice	130 (50.8)	36 (22.0)	<.001
Nystagmus/abnormal eye movements	13 (5.1)	2 (1.2)	.111
Muscle twitching	9 (3.5)	3 (1.8)	.312
Loss of consciousness	9 (3.5)	1 (0.6)	.057
Convulsion	21 (8.2)	12 (7.3)	.742
Opisthotonos/abnormal posturing∗∗	6 (3.1)	4 (3.6)	.803
Acute/flaccid paralysis∗∗	2 (1.0)	0 (0.0)	.284
**Echocardiogram findings**			
Normal echocardiogram	207 (80.9)	140 (85.9)	.183
Abnormal echocardiogram	49 (19.1)	23 (14.0)	.183
Abnormality compatible with thiamine deficiency	31 (63.3)	9 (39.1)	.024
Abnormality not compatible with thiamine deficiency	18 (36.7)	14 (60.9)	.024
**Cranial ultrasound findings**			
Normal cranial ultrasound	93 (36.6)	95 (58.3)	<.001
Abnormal cranial ultrasound	161 (63.4)	68 (41.7)	<.001
Abnormality probably compatible with thiamine deficiency	102 (63.3)	19 (27.9)	<.001
Abnormality possibly compatible with thiamine deficiency	45 (28.0)	19 (27.9)	<.001
Abnormality not compatible with thiamine deficiency	14 (8.7)	30 (44.1)	<.001
**Laboratory parameters**			
ETKac	1.27 (1.14, 1.59)	1.22 (1.08, 1.44)	.017
ETKac >1.25	71 (53.0)	45 (44.6)	.201
Whole blood ThDP (nmol/L)	64.1 (39.9, 89.9)	71.7 (50.3, 111.2)	.072
ThDP <95 nmol/L	118 (78.2)	84 (69.4)	.102
Lactate (mmol/L)	2.2 (1.6, 3.5)	1.9 (1.4, 2.5)	<.001
Lactate >4 mmol/L	48 (20.6)	13 (8.8)	.002
AGP (g/L)	0.78 (0.57, 1.20)	1.05 (0.69, 1.61)	<.001
AGP >1 g/L	79 (33.6)	76 (51.0)	.001
CRP (mg/L)	3.87 (0.60, 17.10)	7.09 (1.36, 30.0)	.004
CRP >5 mg/L	110 (46.8)	85 (57.1)	.051
Hemoglobin (g/L)	10.9 ± 1.8	10.5 ± 2.1	.047
Anemia (hemoglobin <110 g/L)	128 (52.9)	95 (60.1)	.154
Human milk total thiamine (μg/L)††	82.1 (41.3, 139.8)	99.5 (67.2, 147.5)	.034
Human milk total thiamine <200 μg/L	200 (85.8)	118 (88.7)	.432

*AGP*, alpha-1-acid-glycoprotein; *CRP*, C-reactive protein.

∗ Values are n (%), median (Q1, Q3) or mean ± SD; sample size for different assessments may vary (n = 235 children with thiamine biomarkers assessed). Group differences tested via χ² or Kruskal-Wallis tests as appropriate.

† Eligibility criteria for enrollment into study.

‡ n = 327 observed.

§ Respiratory rate >60/min for 3-8 weeks; >50/min for 2-11 months; >40/min for 12-18 months.

¶ Heart rate >160/min for <12 months; >120/min for 12-18 months.

∗∗ n = 121 (26.9%) not recorded.

†† Human milk total thiamine concentrations were calculated as: free thiamine + (thiamine monophosphate x 0.871) + (thiamine diphosphate x 0.707).

Children with and without TRD were hospitalized for a median (Q1, Q3) of 3 (2, 6) days and 4 (2, 7) days, respectively (Table IV, online; available at www.jpeds.com; *P* = .004). The hospital physicians treating the children diagnosed respiratory disorders as the primary diagnosis in 166 children (39.5%) and beriberi as the primary diagnosis in 127 children (30.2%). The primary diagnosis of beriberi was more common among children classified as TRD (n = 101, 39.5%) vs non-TRD (n = 26, 15.9%; *P* < .001). One hundred eighty-six children (44.3%) had a secondary diagnosis of beriberi (44.1% TRD vs 44.5% non-TRD; *P* = .94). Antibiotics and supplemental oxygen were the most frequently administered medications and treatments. No adverse events were reported following thiamine administration, except for 2 children with temporary lethargy within 30 minutes of the injection and 2 children with redness at the injection site.

Among all children with thiamine biomarkers assessed (n = 235), median (Q1, Q3) ETKac was 1.25 (1.11, 1.48), and whole blood ThDP was 66.9 (41.3, 96.1) nmol/L. ETKac was significantly different between children classified as TRD and non-TRD (Table III; 1.27 (1.14, 1.59) and 1.22 (1.08, 1.44) respectively; *P* = .017), although the proportion of children with ETKac >1.25, indicating a high risk of thiamine deficiency did not differ (*P* = .20). Whole blood ThDP was not significantly different among TRD compared with non-TRD children (Table III; 64.1 (39.9, 89.9) vs 71.7 (50.3, 111.2) nmol/L; *P* = .072). Median (Q1, Q3) human milk total thiamine concentration was lower among mothers of children classified as TRD compared with non-TRD (Table III; 82.1 (41.3, 139.8) vs 99.5 (67.2, 147.5) μg/L; *P* = .03), although the proportion of mothers with human milk total thiamine <200 μg/L did not differ between TRD and non-TRD (85.8% vs 88.7%; *P* = .43). Lactate concentration and elevated lactate was significantly higher among TRD children than non-TRD children. In contrast, alpha-1-acid-glycoprotein and C-reactive protein were both lower among TRD compared with non-TRD cases (Table III).

In the TRD cohort, 49 children (19.1%) had abnormal echocardiograms (Table III). Of these abnormal echocardiograms, 31 (63.3%) were compatible with thiamine deficiency. Echocardiograms were compatible with thiamine deficiency if there was left atrial or left ventricular dilation, decreased right or left ventricular systolic function, or hyperdynamic systolic function (suggesting a high cardiac output state). In the non-TRD cohort, 23 children (14.0%) had abnormal echocardiograms. Only 9 (39.1%) of these abnormal studies were compatible with thiamine deficiency which was significantly different from the TRD cohort (*P* = .024). Of the abnormal echocardiograms, there were no significant differences between TRD and non-TRD cohorts in time to normal echocardiogram study at 24 and 48 hours, measures of left ventricular systolic performance, or presence of pericardial effusion (Table V, online; available at www.jpeds.com).

Among children classified as TRD, 161 children (63.1%) had an abnormal cranial ultrasound (Table III). Of these abnormal ultrasounds, 102 (63.3%) were compatible with thiamine deficiency (ie, echogenic putamen and/or caudate nucleus), and a further 45 (28.0%) were possibly compatible with thiamine deficiency. In the non-TRD cohort, 68 (41.7%) ultrasounds were considered abnormal, of which 19 (27.9%) were compatible with thiamine deficiency, and 19 (27.9%) possibly compatible with thiamine deficiency. The difference between cohorts in abnormal cranial ultrasounds compatible with thiamine deficiency was statistically significantly (*P* < .001).

Variables applicable in low resource settings that were selected as most predictive by the model, in order of importance, were hoarse voice/loss of voice, exclusive/predominant breastfeeding, cyanosis, child does not make eye contact and no diarrhea in the previous 2 weeks (Table VI). The AUROC (95% CI) was 0.82 (0.78, 0.86), indicating a high performing model. The cross-validated AUROC (95% CI) was 0.78 (0.63, 0.90), and the model also performed well when applied within specific age strata (<3 months, 3-6 months, and >6 months; AUROC of 0.79-0.84). The high resource model retained the same predictors as the low resource model, except that cranial ultrasounds helped to discriminate TRD cases and no diarrhea in the previous 2 weeks was no longer useful. The AUROC (95% CI) of the high resource model, not including thiamine biomarkers, was 0.84 (0.80, 0.88), again indicating a high performing model.

**Table VI:** Selected predictors of thiamine responsive disorder and relative risks for the low resource and high resource setting models

Predictor	AUROC (95% CI)	RR (95% CI)∗
**Low resource model**	0.82 (0.78, 0.86)	
Hoarse voice/loss of voice		1.58 (1.36, 1.83)
Exclusively or predominantly breastfed		1.99 (1.49, 2.66)
Cyanosis		1.44 (1.22, 1.69)
Does not make eye contact		1.39 (1.20, 1.62)
No diarrhea in the previous 2 weeks		1.59 (1.00, 2.54)
**High resource model**†	0.84 (0.80, 0.88)	
Hoarse voice/loss of voice		1.58 (1.36, 1.83)
Cranial ultrasound		1.50 (1.24, 1.80)
Exclusively or predominantly breastfed		1.99 (1.49, 2.66)
Does not make eye contact		1.39 (1.20, 1.62)

*RR*, relative risk.

∗ Relative risks from bivariate modified Poisson regression to quantify the increased risk of TRD associated with each predictor.

† Not including thiamine biomarkers.

The AUROCs and selected predictors for the various sensitivity analyses are shown in Table VII, online; available at www.jpeds.com. The models that: (1) included children who died within 72 hours of enrollment as non-TRD, (2) excluded children who received thiamine prior to arrival at hospital, and (3) excluded children not assigned a consensus TRD classification after initial pediatrician review, saw minimal drops in performance. Overall, the models performed well across all scenarios. Hoarse voice/loss of voice and predominant breastfeeding were consistently selected across all variations of the model, while cyanosis and no eye contact were selected by three of the four model scenarios. The high resource model including thiamine biomarkers selected ETKac as an additional predictor.

## Discussion

Accurate and timely identification and treatment of TRD is required to prevent the severe adverse events associated with thiamine deficiency. We sought to develop a predictive model for TRD to help reduce these uncertainties and act as a clinical guide to determine when thiamine should be administered in high-risk regions. Considering only variables that are available to clinicians at the time of hospital admission in a low resource setting, among infants and young children hospitalized for signs and symptoms suggestive of TDD, variables selected as most predictive of TRD by the model, in order of importance, were hoarse voice/loss of voice, exclusive/predominant breastfeeding, cyanosis, no eye contact, and no diarrhea in the previous 2 weeks. Consistent predictors were selected in the high resource model, except that cranial ultrasounds helped to discriminate TRD cases.

The majority of children presented with signs and symptoms of respiratory and cardiac distress, namely difficulty breathing, tachypnea, and tachycardia, resembling the cardiac form of TDD described in young infants,^1^ typically peaking at 2-4 months of age and documented in various geographic regions, including South^6^ and Southeast Asia^8^^,^^9^, and to a lesser extent, Africa.^27^ Although few children (<10%) in the current study presented with neurological manifestations of TDD, in contrast to reports of 20%-80% of hospitalized infants in India,^28^^,^^29^ over one-third had cranial abnormalities consistent with thiamine deficiency on ultrasonography. The cranial ultrasound findings suggested thiamine deficiency effects were confined to the basal ganglia. Symmetrically increased echogenicity was seen within the putamina and also in some patients within the caudate nuclei and/or thalamus.^30^ The high rate of abnormalities on cranial ultrasonography even among children determined to be non-TRD or those without clinical features directly referable to the central nervous system indicates that early brain lesions may be present before overt clinical signs of TDD manifest, pointing to the urgent need for public health interventions to prevent thiamine deficiency in early life.

Although many children presented with signs and symptoms resembling the cardiac form of TDD, relatively few patients in the TRD cohort had abnormal echocardiograms. This may be explained by the study design to simplify the echocardiograms for the local physicians who were less experienced with the technique and by the definition of abnormal echocardiograms that was applied in the present study. The echocardiograms in the study focused on basic, proven, and highly reproducible measurements of ventricular systolic function. It is possible that cardiac systolic performance as measured by echocardiogram may not be sensitive enough to identify patients with TRD. Additionally, cardiac symptoms of TDD may not correlate directly to ventricular systolic function. Other, more sensitive, echocardiographic measures may need to be considered in future studies, such as left ventricle chamber dilation, mitral valve regurgitation, or signs of pulmonary hypertension.

The development of a predictive model for TRD was complicated by the fact that clinical responsiveness to thiamine administration could not be isolated from other interventions. Response to thiamine is often described as rapid,^2^ thus in the current study thiamine responsiveness was judged as an improvement in vital signs and clinical symptoms within 8-12 hours, based on what is known about how quickly children with cardiac beriberi respond to thiamine. However, in the present study, most children not only received thiamine but other medications and supportive care, so it was difficult to judge whether an infant responded to the thiamine treatment alone and/or other treatments. Moreover, children with both TDD and other diseases, such as respiratory infections that can produce similar symptoms, may not have demonstrated a clear response to thiamine because of a slower resolution of other diseases. These issues could be disentangled only through the inclusion of a placebo group, which is not feasible for ethical reasons. As 95% of children in the present study with signs and symptoms consistent with TDD were judged as having some responsiveness to thiamine (only 5.4% were classified as not likely TRD), a predictive model for TRD would likely not be useful as a treatment tool in a setting such as Luang Prabang with a high rate of TRD. The lack of rapid diagnostic tests, the fact that thiamine is inexpensive, safe, effective, and easy to administer, and the high risk of mortality associated with missed treatment, justifies wide-scale thiamine administration to children with clinical features suggestive of possible TDD in high risk settings. Therefore, our recommendation is to provide thiamine to all children presenting with possible TDD in this and similar high risk settings.

The selected predictors of TRD (hoarse voice/loss of voice, exclusive or predominant breastfeeding, cyanosis, not making eye contact, and no diarrhea in the previous 2 weeks) are consistent with expectations based on clinical experience and published literature,^2^ and for the most part did not vary across sensitivity analyses, with good model performance across all variations of the model. It is important to remember that the selected predictors are among children already selected as having signs and symptoms suggestive of TDD. While symptoms of respiratory and cardiac distress were the most prevalent among the hospitalized infants and significantly higher among the TRD children, these were not selected by the model as incrementally predictive of TRD, when adjusting for variables already in the model. In the high resource model, cranial ultrasounds were selected as being very useful in discriminating TRD against non-TRD. Point-of-care ultrasound use in low resource settings has expanded in recent years because of their high utility, low cost, and easy maintenance.^31^^,^^32^ Although ETKac did add predictive value to the high resource model, the wide range and small difference, although statistically significant, in ETKac between TRD and non-TRD cohorts, adds little clinical value. The use of biomarkers for thiamine deficiency and their association with clinical symptoms of TDD have been inconsistent,^8^^,^^9^^,^^33^ and further research is needed to better understand the relationship between biomarkers of thiamine status and clinical manifestations of TDD.

Acute infectious illnesses are common among infants and young children in Lao People’s Democratic Republic and could present in a similar way to TDD. Acute illness might unmask previously latent TDD or low thiamine status may predispose children to more severe illness.^34^^,^^35^ In the present study, non-TRD children had significantly higher alpha-1-acid-glycoprotein and C-reactive protein (of which the pediatrician panel were unaware when determining TRD status) than TRD children, suggesting non-TRD children had more severe or multiple infections. Conversely, lactate was significantly higher among TRD compared with non-TRD children. Elevated lactate levels have been suggested as a nonspecific indicator of metabolic imbalance associated with thiamine deficiency, and thiamine deficiency should be considered in cases of otherwise unexplained hyperlactatemia.^36^

There are currently no evidence-based recommendations or guidelines regarding the thiamine dosage regimen and duration of treatment for infants and young children with TDD, and doses used in clinical practice vary widely (50-1500 mg/day).^2^ The World Health Organization recommendations from 1999 for treating infantile thiamine deficiency,^37^ and specifically for the Western Pacific region,^38^ suggest immediate treatment with 25-50 mg IV/IM thiamine followed by daily oral doses of 3-10 mg thiamine for 2-6 weeks. In the present study, a daily dose of 25-50 mg/day was considered potentially insufficient based on local clinical experience, and a daily dose of 100 mg thiamine was determined most appropriate for as long as deemed necessary according to the child's clinical condition, in accordance with standard treatment procedures for infants presenting with TDD at the hospital where the study was conducted. Further research is needed to optimize treatment regimens, including parenteral vs oral treatment of various doses and durations for differing clinical presentations of TDD.

Strengths of the study include the large sample size and extensive data collection based on physical examinations, ultrasonography, and biomarker data among infants and young children with clinical signs and symptoms suggestive of TDD. The TRD status was derived from the full dataset, including known risk factors such as exclusive/predominant breastfeeding and clinical signs and symptoms at hospital admission. The predictive model incorporated these variables and so is not solely data-driven, but instead builds on the previous knowledge and experience of the pediatrician panel. A possible limitation of the study is the use of an off-site pediatrician panel to review case reports to define TRD. While remote diagnosis allowed for the input of pediatricians with extensive expertise in beriberi, their diagnosis was based on their interpretation of the case reports rather than direct patient contact and was dependent on their prior clinical experiences. Nevertheless, the high level of reviewer agreement across the pediatrician panel provides confidence in the predictive model for TRD and the case review process was reliable and consistently applied. A further limitation of the present study was that the clinical treatments and discharge diagnosis assigned by the treating hospital physicians, were subject to large variability as the hospital physicians were not standardized in these domains. However, these factors were only a small part of the larger dataset, and the pediatricians comprising the study panel considered the complete set of data available to them. Lastly, it must be emphasized that the predictive model for TRD has to be considered within the context of children already presenting with signs and symptoms suggestive of TDD and may be context specific. This may limit generalizability of the findings to other settings. Thus, the predictive model requires assessment and validation in other contexts globally with varying degrees of thiamine deficiency. Although the TRD predictive model may not be useful as a treatment tool in the local context where the study was conducted, it may be useful for other purposes such as sentinel surveillance or as a clinical tool to determine the risk of TRD in areas with lower prevalence of thiamine deficiency. Although the eligibility criteria were developed based on TDD compatible symptoms in the broadest sense, there may be other currently unrecognized signs and symptoms of TRD among infants and young children.

In conclusion, among infants and young children hospitalized with signs and symptoms suggestive of TDD, variables selected as most predictive of TRD were hoarse voice/loss of voice, exclusive/predominant breastfeeding, cyanosis, no eye contact, and no diarrhea in the previous 2 weeks, as well as cranial ultrasonography and ETKac when included in the high resource model. While these specific features were identified as most predictive of TRD, the high prevalence of TRD in this population, suggests that all children presenting with clinical signs and symptoms suggestive of TDD in this and similar high risk settings should be treated with thiamine due to the high risk associated with missed treatment. The usefulness of the predictive model in other contexts requires further exploration.

## Data Statement

The data that support the findings of this study are openly available in Open Science Framework: https://osf.io/jfke3/.

## CRediT Authorship Contribution Statement

**Taryn J. Smith:** Writing – original draft, Visualization, Supervision, Project administration, Methodology, Investigation. **Charles D. Arnold:** Writing – review & editing, Validation, Methodology, Formal analysis, Data curation, Conceptualization. **Philip R. Fischer:** Writing – review & editing, Methodology, Conceptualization. **Indi Trehan:** Writing – review & editing, Methodology, Conceptualization. **Laurent Hiffler:** Writing – review & editing, Methodology, Conceptualization. **Dalaphone Sitthideth:** Writing – review & editing, Supervision, Project administration, Investigation. **Rebecca Stein-Wexler:** Writing – review & editing, Methodology. **Jay Yeh:** Writing – review & editing, Methodology. **Kerry S. Jones:** Writing – review & editing, Resources. **Daniela Hampel:** Writing – review & editing, Resources. **Daniel J. Tancredi:** Writing – review & editing, Methodology, Formal analysis. **Michael A. Schick:** Writing – review & editing, Methodology. **Christine N. McBeth:** Writing – review & editing, Methodology. **Xiuping Tan:** Writing – review & editing, Software, Data curation. **Lindsay H. Allen:** Writing – review & editing, Resources. **Somphou Sayasone:** Writing – review & editing, Resources. **Sengchanh Kounnavong:** Writing – review & editing, Supervision, Project administration, Conceptualization. **Sonja Y. Hess:** Writing – review & editing, Supervision, Project administration, Methodology, Funding acquisition, Conceptualization.

## Declaration of Competing Interest

This work was supported by the 10.13039/100000865Bill & Melinda Gates Foundation [grant number INV-009736]. Under the grant conditions of the Foundation, a Creative Commons Attribution 4.0 Generic License has already been assigned to the Author Accepted Manuscript version that might arise from this submission. Kenneth Brown provided technical support in the study design while working for the Bill & Melinda Gates Foundation. The sponsor had no role in the data collection, analysis, interpretation, or writing of this article.

The spouse of S.Y.H. previously worked for and currently consults for the Bill & Melinda Gates Foundation. DJT has provided statistical consultations to International Flavors and Fragrances Inc. All other authors declare that they have no conflicts of interest.

This research was supported by the NIHR Cambridge Biomedical Research Centre (NIHR203312). The views expressed are those of the authors and not necessarily those of the NIHR or the Department of Health and Social Care.

## Supplementary Data

STROBE_checklist_cohort

Data Statement

Table I

Table IV

Table V

Table VII

## References

[1] Whitfield K.C., Bourassa M.W., Adamolekun B., Bergeron G., Bettendorff L., Brown K.H. (2018). Thiamine deficiency disorders: diagnosis, prevalence, and a roadmap for global control programs. Ann N Y Acad Sci.

[2] Smith T.J., Johnson C.R., Koshy R., Hess S.Y., Qureshi U.A., Mynak M.L. (2021). Thiamine deficiency disorders: a clinical perspective. Ann N Y Acad Sci.

[3] Rao S.N., Chandak G.R. (2010). Cardiac beriberi: often a missed diagnosis. J Trop Pediatr.

[4] Qureshi U.A., Sami A., Altaf U., Ahmad K., Iqbal J., Wani N.A. (2016). Thiamine responsive acute life threatening metabolic acidosis in exclusively breast-fed infants. Nutrition.

[5] Bhat J.I., Rather H.A., Ahangar A.A., Qureshi U.A., Dar P., Ahmed Q.I. (2017). Shoshin beriberi-thiamine responsive pulmonary hypertension in exclusively breastfed infants: a study from northern India. Indian Heart J.

[6] Sastry U.M.K., Mxx J., Kumar R.K., Ghosh S., Bharath A.P., Subramanian A. (2021). Thiamine-responsive acute severe pulmonary hypertension in exclusively breastfeeding infants: a prospective observational study. Arch Dis Child.

[7] Luxemburger C., White N.J., ter Kuile F., Singh H.M., Allier-Frachon I., Ohn M. (2003). Beri-beri: the major cause of infant mortality in Karen refugees. Trans R Soc Trop Med Hyg.

[8] Coats D., Shelton-Dodge K., Ou K., Khun V., Seab S., Sok K. (2012). Thiamine deficiency in Cambodian infants with and without beriberi. J Pediatr.

[9] Porter S.G., Coats D., Fischer P.R., Ou K., Frank E.L., Sreang P. (2014). Thiamine deficiency and cardiac dysfunction in cambodian infants. J Pediatr.

[10] Soukaloun D., Kounnavong S., Pengdy B., Boupha B., Durondej S., Olness K. (2003). Dietary and socio-economic factors associated with beriberi in breastfed Lao infants. Ann Trop Paediatr.

[11] Barennes H., Sengkhamyong K., René J.P., Phimmasane M. (2015). Beriberi (thiamine deficiency) and high infant mortality in northern Laos. PLoS Negl Trop Dis.

[12] Allen L.H. (2012). B vitamins in breast milk: relative importance of maternal status and Intake, and effects on infant status and function. Adv Nutr.

[13] Hiffler L., Rakotoambinina B., Lafferty N., Martinez Garcia D. (2016). Thiamine deficiency in tropical pediatrics: New insights into a neglected but vital metabolic challenge. Front Nutr.

[14] Fattal-Valevski A., Azouri-Fattal I., Greenstein Y.J., Guindy M., Blau A., Zelnik N. (2009). Delayed language development due to infantile thiamine deficiency. Dev Med Child Neurol.

[15] Harel Y., Zuk L., Guindy M., Nakar O., Lotan D., Fattal-Valevski A. (2017). The effect of subclinical infantile thiamine deficiency on motor function in preschool children. Matern Child Nutr.

[16] Hess S.Y., Smith T.J., Fischer P.R., Trehan I., Hiffler L., Arnold C.D. (2020). Establishing a case definition of thiamine responsive disorders among infants and young children in Lao PDR: protocol for a prospective cohort study. BMJ Open.

[17] Lu J., Frank E.L. (2008). Rapid HPLC measurement of thiamine and its Phosphate Esters in whole blood. Clin Chem.

[18] Jones K.S., Parkington D.A., Cox L.J., Koulman A. (2021). Erythrocyte transketolase activity coefficient (ETKAC) assay protocol for the assessment of thiamine status. Ann N Y Acad Sci.

[19] Institute of Medicine (1998).

[20] Turck D., Bresson J.-L., Burlingame B., Dean T., Fairweather-Tait S., EFSA Panel on Dietetic Products Nutrition and Allergies (2016). Dietary reference values for thiamin. EFSA J.

[21] Erhardt J.G., Estes J.E., Pfeiffer C.M., Biesalski H.K., Craft N.E. (2004). Combined measurement of Ferritin, Soluble Transferrin Receptor, Retinol Binding protein, and C-reactive protein by an inexpensive, sensitive, and Simple Sandwich enzyme-linked immunosorbent assay technique. J Nutr.

[22] Hampel D., York E.R., Allen L.H. (2012). Ultra-performance liquid chromatography tandem mass-spectrometry (UPLC–MS/MS) for the rapid, simultaneous analysis of thiamin, riboflavin, flavin adenine dinucleotide, nicotinamide and pyridoxal in human milk. J Chromatogr B Analyt Technol Biomed Life Sci.

[23] Cashin K., Oot L. (2018). *Food and Nutrition technical Assistance III Project*.

[24] Hess S.Y., Smith T.J., Arnold C.D. (2019). https://osf.io/jfke3/.

[25] Steyerberg E.W., Vergouwe Y. (2014). Towards better clinical prediction models: seven steps for development and an ABCD for validation. Eur Heart J.

[26] Smith T.J., Tan X., Arnold C.D., Sitthideth D., Kounnavong S., Hess S.Y. (2022). Traditional prenatal and postpartum food restrictions among women in northern Lao PDR. Matern Child Nutr.

[27] Hiffler L., Escajadillo K., Rocaspana M., Janet S. (2020). Acute respiratory failure in an infant and thiamine deficiency in West Africa: a case report. Oxf Med Case Reports.

[28] Bhat J.I., Ahmed Q.I., Ahangar A.A., Charoo B.A., Sheikh M.A., Syed W.A. (2017). Wernicke's encephalopathy in exclusive breastfed infants. World J Pediatr.

[29] Qureshi U.A., Bhat A.S., Qureshi U., Ahmad K., Wani N.A., Bashir A. (2021). Infantile thiamine deficiency: Redefining the clinical patterns. Nutrition.

[30] Douglass K., Stein-Wexler R., Schick M.A., Stein-Wexler R., Nasirishargh A. (2020). Ultrasound in resource-limited settings: a case based, open access text.

[31] Nelson B.P., Melnick E.R., Li J. (2011). Portable ultrasound for remote Environments, part I: Feasibility of field deployment. J Emerg Med.

[32] Stewart K.A., Navarro S.M., Kambala S., Tan G., Poondla R., Lederman S. (2020). Trends in ultrasound use in low and middle income Countries: a systematic review. Int J MCH AIDS.

[33] Keating E.M., Nget P., Kea S., Kuong S., Daly L., Phearom S. (2015). Thiamine deficiency in tachypnoeic cambodian infants. Paediatr Int Child Health.

[34] Khounnorath S., Chamberlain K., Taylor A.M., Soukaloun D., Mayxay M., Lee S.J. (2011). Clinically Unapparent infantile thiamin deficiency in Vientiane, Laos. PLoS Negl Trop Dis.

[35] Kauffman G., Coats D., Seab S., Topazian M.D., Fischer P.R. (2011). Thiamine deficiency in ill children. Am J Clin Nutr.

[36] Andersen L.W., Mackenhauer J., Roberts J.C., Berg K.M., Cocchi M.N., Donnino M.W. (2013). Etiology and therapeutic approach to elevated lactate levels. Mayo Clin Proc.

[37] World Health Organization (1999).

[38] World Health Organization Western Pacific Region (2017).

