# Supplementary material for: A Predictive Model for Thiamine Responsive Disorders Among Infants and Young Children: Results from a Prospective Cohort Study in Lao People's Democratic Republic
Source: J Pediatr. 2024 May;268:113961. doi: 10.1016/j.jpeds.2024.113961 (PMC11092315; doi:10.1016/j.jpeds.2024.113961)
Supplement: Data Statement [file mmc2.docx]

Data sharing statement: The data that support the findings of this study are openly available at <https://osf.io/jfke3/>.
