# Supplementary material for: A Predictive Model for Thiamine Responsive Disorders Among Infants and Young Children: Results from a Prospective Cohort Study in Lao People's Democratic Republic
Source: J Pediatr. 2024 May;268:113961. doi: 10.1016/j.jpeds.2024.113961 (PMC11092315; doi:10.1016/j.jpeds.2024.113961)
Supplement: Table I [file mmc3.docx]

**Table 1.** Reviewer agreements with consensus TRD status and low-resource setting model

|  | **Consensus** | **Reviewer 1** | **Reviewer 2** | **Reviewer 3** |
| --- | --- | --- | --- | --- |
| **TRD status** |  |  |  |  |
| Classic beriberi | 92 (21.8) | 117 (27.4) | 166 (38.9) | 33 (7.7) |
| Probable TRD | 165 (39.0) | 149 (34.9) | 94 (22.0) | 188 (44.0) |
| Possible TRD | 143 (33.8) | 127 (29.7) | 123 (28.8) | 181 (42.4) |
| Not likely TRD | 23 (5.4) | 34 (8.0) | 44 (10.3) | 25 (5.9) |
| **AUROC (95% CI)** | 0.82 (0.78, 0.86) | 0.74 (0.69, 0.79) | 0.78 (0.74, 0.83) | 0.78 (0.74, 0.83) |

Values are n (%)
