# Supplementary material for: A Predictive Model for Thiamine Responsive Disorders Among Infants and Young Children: Results from a Prospective Cohort Study in Lao People's Democratic Republic
Source: J Pediatr. 2024 May;268:113961. doi: 10.1016/j.jpeds.2024.113961 (PMC11092315; doi:10.1016/j.jpeds.2024.113961)
Supplement: Table IV [file mmc4.docx]

**Table 4.** Final primary and secondary diagnoses by treating hospital physicians treating at discharge and medications and adverse events by TRD status determined by a pediatrician panel (n = 420)*

|  | **TRD**  **(n = 256)** | **Non-TRD**  **(n = 164)** | ***P* value** |
| --- | --- | --- | --- |
| Duration of stay in hospital (days)^†^ | 3 (2, 6) | 4 (2, 7) | 0.004 |
| **Primary diagnosis** |  |  |  |
| Beriberi | 101 (39.5) | 26 (15.9) | <0.001 |
| Respiratory disorders |  |  |  |
| Pneumonia | 33 (12.9) | 29 (17.7) | 0.177 |
| Viral lower respiratory infection | 53 (20.7) | 34 (20.7) | 0.994 |
| Viral upper respiratory infection | 4 (1.6) | 3 (1.8) | 0.835 |
| Asthma | 3 (1.2) | 2 (1.2) | 0.965 |
| Pertussis | 3 (1.2) | 2 (1.2) | 0.965 |
| Infection |  |  |  |
| Sepsis | 14 (5.5) | 7 (4.3) | 0.582 |
| Urinary tract infection | 0 (0) | 2 (1.2) | 0.152 |
| Gastrointestinal infection |  |  |  |
| Diarrhea | 9 (3.5) | 7 (4.3) | 0.939 |
| Vomiting | 0 (0) | 2 (1.2) | 0.152 |
| Dysentery | 1 (0.4) | 0 (0) | 0.999 |
| Central nervous system disorder |  |  |  |
| Meningitis (including meningoencephalitis) | 13 (5.1) | 7 (4.3) | 0.704 |
| Seizure | 1 (0.4) | 4 (2.4) | 0.079 |
| Cardiovascular disorder | 7 (2.7) | 9 (5.5) | 0.150 |
| Malnutrition | 5 (2.0) | 12 (7.3) | 0.007 |
| Anemia | 0 (0) | 4 (2.4) | 0.023 |
| Developmental delay | 0 (0) | 1 (0.6) | 0.999 |
| Other | 9 (3.5) | 13 (7.9) | 0.045 |
| **Secondary diagnoses^‡^** |  |  |  |
| Beriberi | 113 (44.1) | 73 (44.5) | 0.940 |
| Respiratory disorders | 62 (24.2) | 29 (17.7) | 0.113 |
| Infection | 14 (5.5) | 8 (4.9) | 0.791 |
| Gastrointestinal infection | 12 (4.7) | 8 (4.9) | 0.929 |
| Central nervous system disorder | 7 (2.7) | 8 (4.9) | 0.248 |
| Cardiovascular disorder | 4 (1.6) | 4 (2.4) | 0.521 |
| Developmental delay | 2 (0.8) | 5 (3) | 0.077 |
| Malnutrition | 4 (1.6) | 10 (6.1) | 0.012 |
| Other | 22 (8.6) | 24 (14.6) | 0.053 |
| **Medications and treatments** |  |  |  |
| Number of thiamine doses administered^†^ | 4 (3, 5) | 4 (3, 6) | 0.073 |
| Antibiotics | 156 (60.9) | 117 (71.3) | 0.029 |
| Supplemental oxygen | 171 (66.8) | 93 (56.7) | 0.037 |
| IV fluid or medication | 130 (50.8) | 72 (43.9) | 0.169 |
| CPAP | 82 (32) | 45 (27.4) | 0.317 |
| Nasogastric feeding tube | 77 (30.1) | 61 (37.2) | 0.130 |
| Anticonvulsant | 26 (10.2) | 12 (7.3) | 0.322 |
| Steroid bronchodilator | 9 (3.5) | 7 (4.3) | 0.694 |
| Antipyretics / analgesics | 25 (9.8) | 23 (14.0) | 0.181 |
| Diuretics | 26 (10.2) | 21 (12.8) | 0.401 |
| Fluid resuscitation | 19 (7.4) | 13 (7.9) | 0.849 |
| Macronutrient supplementation | 10 (3.9) | 6 (3.7) | 0.897 |
| Micronutrient supplementation | 56 (21.9) | 40 (24.4) | 0.649 |
| Anti-infectives (topical/skin) | 13 (5.1) | 12 (7.3) | 0.344 |
| Anti-infectives (systemic) | 2 (0.8) | 5 (3.0) | 0.077 |
| Anti-hypertensives | 3 (1.2) | 3 (1.8) | 0.580 |
| Cardiac medications | 4 (1.6) | 4 (2.4) | 0.521 |
| Respiratory medications | 5 (2.0) | 4 (2.4) | 0.737 |
| Anti-seizure medication | 5 (2.0) | 3 (1.8) | 0.928 |
| Gastrointestinal medications | 6 (2.3) | 4 (2.4) | 0.950 |
| Steroid cream | 0 (0.0) | 2 (1.2) | 0.411 |
| Steroids | 2 (0.8) | 1 (0.6) | 0.839 |
| Surgical interventions | 0 (0.0) | 2 (1.2) | 0.152 |
| **Safety and adverse events** |  |  |  |
| Redness at injection site | 2 (0.8) | 0 (0.0) | 0.999 |
| Swelling at injection site | 0 (0.0) | 0 (0.0) | 0.999 |
| Temporary lethargy following thiamine administration | 2 (0.8) | 0 (0.0) | 0.999 |

^*^ Values are n (%) unless otherwise stated.

^†^ Median (Q1, Q3)

^‡^ May have multiple secondary diagnoses
