# Supplementary material for: A Predictive Model for Thiamine Responsive Disorders Among Infants and Young Children: Results from a Prospective Cohort Study in Lao People's Democratic Republic
Source: J Pediatr. 2024 May;268:113961. doi: 10.1016/j.jpeds.2024.113961 (PMC11092315; doi:10.1016/j.jpeds.2024.113961)
Supplement: Table V [file mmc5.docx]

**Table 5.** Echocardiography findings by TRD status (n = 420)*

|  | **TRD**  **(n = 256)** | **Non-TRD**  **(n = 164)** | ***P* value** |
| --- | --- | --- | --- |
| Abnormal baseline echocardiogram | 49 (19.1) | 23 (14.0) | 0.183 |
| Abnormal baseline echocardiogram compatible with thiamine deficiency | 31 (12.1) | 9 (5.6) | 0.024 |
| Normal at 24 hrs | 15/31 (48.4) | 4/8 (44.4) | 0.935 |
| Normal at 48 hrs | 15/29 (22.6) | 3/8 (33.3) | 0.476 |
| Abnormal baseline echocardiogram, not compatible with thiamine deficiency | 18 (7.0) | 14 (8.5) | 0.728 |
| Normal at 24 hrs | 4/18 (22.2) | 2/13 (15.4) | 0.634 |
| Normal at 48 hrs | 5/16 (31.3) | 2/13 (15.4) | 0.321 |
| EPSS (mm) | 3.5 ± 1.2 | 3.5 ± 1.1 | 0.923 |
| EPSS abnormal (>6 mm) | 6 (2.3) | 2 (1.2) | 0.415 |
| FS (%) | 38.9 ± 7.4 | 38.8 ± 6.8 | 0.999 |
| FS abnormal (<29%) | 3 (1.2) | 3 (1.8) | 0.574 |
| LVEF (%) | 71.5 ± 8.0 | 71.3 ± 7.8 | 0.845 |
| EF abnormal (>55%) | 2 (0.8) | 1 (0.6) | 0.843 |
| TAPSE (mm) | 11.6 ± 2.4 | 12.2 ± 2.3 | 0.011 |
| TAPSE abnormal^†^ | 17 (6.7) | 10 (6.1) | 0.829 |
| Pericardial effusion | 23 (9.0) | 13 (8.0) | 0.711 |

LVEF, left ventricular ejection fraction; EPSS, E-point septal separation; FS, fractional shortening; TAPSE, tricuspid annular plane systolic excursion

^*^ Values are n (%) or mean ± SD

^†^ Abnormal TAPSE: <7 mm for 0-30 days; <8 mm for 1-3 months; <10 mm for 4-6 months; <11 mm for 7-12 months; <12 mm for 13-18 months
