# Supplementary material for: A Predictive Model for Thiamine Responsive Disorders Among Infants and Young Children: Results from a Prospective Cohort Study in Lao People's Democratic Republic
Source: J Pediatr. 2024 May;268:113961. doi: 10.1016/j.jpeds.2024.113961 (PMC11092315; doi:10.1016/j.jpeds.2024.113961)
Supplement: Table VII [file mmc6.docx]

**Table 7.** AUROC and selected predictors of TRD for sensitivity analyses

| **Analysis** | **n** | **Model** | **AUROC** | **Predictors of TRD** |
| --- | --- | --- | --- | --- |
| Including participants who died within 72 hours of enrollment as non-TRD | 439 | Low resource | 0.78 (0.73, 0.82) | Hoarse voice/loss of voice  Exclusive or predominant breastfeeding  No eye contact  Abnormal eye movement  No fever |
| Excluding participants who received thiamine prior to arrival at hospital | 387 | Low resource | 0.81 (0.76, 0.85) | Hoarse voice/loss of voice  Exclusive or predominant breastfeeding  Cyanosis |
| Excluding participants not assigned a consensus TRD classification after initial pediatrician panel review | 365 | Low resource | 0.81 (0.77, 0.86) | Hoarse voice/loss of voice  Exclusive or predominant breastfeeding  Cyanosis  No eye contact |
| High resource model including thiamine biomarkers | 261 | High resource | 0.84 (0.80, 0.89) | Hoarse voice/loss of voice  Exclusive or predominant breastfeeding  ETKac  Cranial ultrasound  Cyanosis |
